# Analysis of MDA, SOD, TAOC, MNCV, SNCV, and TSS scores in patients with diabetes peripheral neuropathy

**DOI:** 10.1515/biol-2022-0945

**Published:** 2024-10-29

**Authors:** Yukun Jia, Yan Li

**Affiliations:** Department of Endocrinology, Tangshan Gongren Hospital, Tangshan, 063000, China

**Keywords:** traditional Chinese medicine package, red light irradiation, lipoic acid, DPN, TSS score, MNCV, SNCV, MDA, SOD, TAOC

## Abstract

To explore the impact of score in patients with diabetes peripheral neuropathy (DPN) treated with traditional Chinese medicine package (TCMP) plus red light therapy and lipoic acid on malondialdehyde (MDA), erythrocyte superoxide dismutase (SOD), total antioxidant capacity (TAOC), motor nerve conduction velocity (MNCV), sensory nerve conduction velocity (SNCV), and Toronto Clinical Scoring System (TSS). A total of 108 patients with DPN hospitalized in the hospital were chosen and divided into groups with the random number table. In the control group (CG) 54 patients were treated with conventional lipoic acid, and 54 patients in the experimental group (EG) accepted TCMP plus red light on the basis of the CG. The MDA, SOD, TAOC, MNCV, SNCV, and TSS scores before treatment and after treatment were compared between the two groups. Before treatment, there was no statistically significant difference in the levels of oxidation indicators, nerve conduction velocity, and symptom scores between the two groups (*P* > 0.05). After treatment, the MDA in the EG was lower than that in the CG, with a statistical significance difference (*P* < 0.05). The SOD and TAOC in the EG were higher than those in the CG, and the difference was statistically significant (*P* < 0.05). The MNCV and SNCV of median nerve, common peroneal nerve, and tibial nerve in the EG were significantly higher than those in the CG (*P* < 0.05). The TSS score of the EG was lower than that of the CG, and the difference was statistically significant (*P* < 0.05). The treatment of patients with DPN with lipoic acid plus TCMP and red light therapy can improve the symptoms and signs of disease, promote the recovery of motor and sensory conduction velocity, and optimize the body oxidation indicators.

## Graphical abstract

**Figure:**
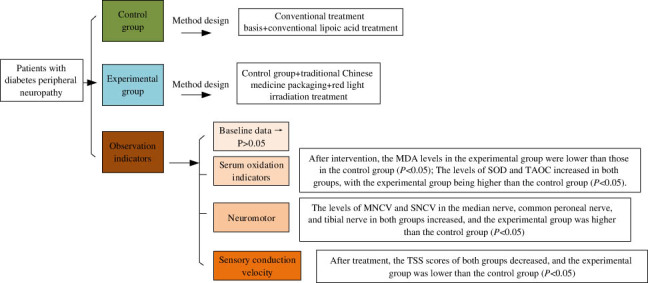

## 1 Introduction

Epidemiological survey in 2019 showed that 9.3% of patients with diabetic mellitus (DM) were diagnosed globally. Among them, the domestic incidence rate can reach 28.5% in the global incidence rate, which is the highest incidence rate in the world [1]. Diabetes peripheral neuropathy (DPN) is one of the high incidence complications of DM. During the course of DM, the incidence rate of DPN can reach 90% within 10 years, and it shows a significant trend of increasing year-by-year [2]. The occurrence of DPN not only leads to physical and limb dysfunction, but in severe cases, it can even lead to foot ulcers, increasing the incidence of gangrene and disability. This can threaten the health of the body and reduce its quality of life. Therefore, improving the treatment of DPN is an urgent issue that needs to be addressed in clinical practice. Currently, clinical treatment of DPN mainly utilizes antioxidants. Medications can improve the microcirculation of the body, alleviate symptoms of tissue nerve hypoxia, promote motor and sensory conduction velocity, and thereby reduce the damage of diseases to neural function. Lipoic acid is a common antioxidant that acts on tissues and can significantly inhibit lipid peroxidation. At the same time, it can increase the activity of nerve Na^+^−K^+^-ATPase and improve the body’s nerve conduction velocity. Drugs have good therapeutic effects on diseases, but the efficacy of some patients in the treatment is still not ideal [3]. The most critical factor that causes DPN is that diabetes patients are in a state of vein blockage for a long time, which leads to clinical symptoms such as fatigue, weakness, decreased resistance, palpitations, incontinence, etc. In traditional Chinese medicine treatment methods, medicinal herbs such as *Achyranthes bidentata* and *Angelica sinensis* have obvious effects on removing blood stasis, dispelling dampness, warming yang, and unblocking collaterals, which can effectively help patients alleviate and eliminate a series of clinical symptoms. For example, Ai et al. [4] explored the mechanism of Huangqi Guizhi Wuwu Decoction in the treatment of DPN with the help of the platform of systematic pharmacology analysis of traditional Chinese medicine, and collected cross targets to build a database. The results indicated that Quercetin and Kaempferol in Guizhi Wuwu Decoction can effectively target proteins and regulate signaling pathways. Apiaceae medicinal plants can be used to treat diseases such as central nervous system, cardiovascular system, and respiratory system [5]. At the same time, Santos et al. [6] believed that plant products such as terpenes, flavonoids, and alkaloids have potential anti-injury effects on peripheral neuropathy. The red light therapy device can increase the patient’s pain threshold, improve local tissue blood circulation, alleviate muscle spasms, and achieve therapeutic effects of swelling reduction, pain relief, and muscle growth through the irradiation of instrument red light. As Wang et al. [7] explored the efficacy and safety of low-level phototherapy on DPN, the data on pain relief in patients had a 95% confidence interval and improved overall symptoms, reducing the occurrence of adverse events. Elham et al. [8] found that both single cavity infrared light and low-level laser therapy can effectively improve the activity of deep peroneal nerve neurons, alleviate pain, and improve daily living abilities in patients with DPN. Pharmacological treatment is always associated with limited efficacy and adverse reactions. Lin et al. [9] found that 10.6 μm laser moxibustion wave can alleviate symptoms, improve quality of life, and avoid serious side effects in DPN patients. The red light therapy instrument can increase the patient’s pain threshold, improve local tissue blood circulation, and alleviate muscle spasms by irradiating the instrument with red light. Treatment can achieve therapeutic effects such as swelling reduction, pain relief, and muscle growth, improving patient comfort while also enhancing treatment effectiveness. At present, there are few studies on the use of traditional Chinese medicine package (TCMP) combined with red light therapy to assist lipoic acid in the treatment of DPN. Based on this, this study focuses on DPN patients and analyzes the effectiveness of adjuvant therapy. The following is the report.

## 2 Materials and methods

### 2.1 General information

A total of 108 patients with DPN hospitalized in the hospital from December 2022 to December 2023 were selected for investigation. They were divided into control group (CG) and experimental group (EG) according to random number table, with 54 patients in each group. It needs to compare the gender, age, duration of DM, duration of DPN, glycosylated hemoglobin (HbAlc), fasting plasma glucose (FPG), human total cholesterol (TC), triglyceride (TG), high-density lipoprotein (HDL), and low-density lipoprotein (LDL) between the two groups. It has no statistical significance difference (*P* > 0.05), as denoted in Table 1.

**Table 1:** General information of two groups (*x* ± *s*, %)

Group	Number of cases	Gender		Age (years)	Duration of DM (years)	Duration of DPN (years)	HbAlc (%)
		Male	Female				
CG	54	29	25	59.42 ± 5.34	12.48 ± 2.15	6.49 ± 1.64	7.69 ± 1.34
EG	54	31	23	59.81 ± 5.16	12.74 ± 2.03	6.82 ± 1.50	7.82 ± 1.27
*x* ^2^/*t*		0.150	0.386	0.646	1.091	0.430	
*P*		0.699	0.700	0.520	0.278	0.668	

Group	Number of cases	FPG (mmol/L)	TC (mmol/L)	TG (mmol/L)	HDL (mmol/L)	LDL (mmol/L)
CG	54	7.12 ± 0.53	5.11 ± 0.41	1.27 ± 0.20	1.26 ± 0.15	3.26 ± 0.31
EG	54	7.30 ± 0.48	5.02 ± 0.38	1.18 ± 0.27	1.29 ± 0.10	3.17 ± 0.22
*x* ^2^/*t*		1.850	1.183	1.968	1.223	1.740
*P*		0.067	0.239	0.052	0.224	0.085

Note: DM: diabetic mellitus; DPN: diabetes peripheral neuropathy; HbAlc: glycosylated hemoglobin; FPG: fasting plasma glucose; TC: total cholesterol; TG: triglyceride; HDL: high-density lipoprotein; LDL: low-density lipoprotein.


**Informed consent:** Informed consent has been obtained from all individuals included in this study.
**Ethical approval:** The research related to human use has been complied with all the relevant national regulations, institutional policies and in accordance with the tenets of the Helsinki Declaration, and has been approved by the Ethics Committee Tangshan Gongren Hospital.

### 2.2 Inclusion and exclusion criteria

#### 2.2.1 Inclusion criteria

Inclusion criteria included: (a) diagnosed as DM with a course of more than 1 year; (b) comply with the relevant diagnostic criteria of DPN in the 2016 Chinese Medicine Clinical Diagnosis and Treatment Guidelines [10]; (c) significant clinical symptoms, including numbness, pain, and sensory abnormalities; and (d) normal cognitive, reading and writing abilities, and able to cooperate with the implementation of treatment.

#### 2.2.2 Exclusion criteria

Exclusion criteria included: (a) peripheral nerve dysfunction caused by cervical or lumbar diseases or chemotherapy drugs, (b) severe diabetes acidosis, (c) concomitant severe cardiovascular disease or liver and kidney dysfunction, and (d) serum creatinine and transaminase levels increased compared to normal values. The study has been approved and supported by the hospital ethics committee.

### 2.3 Methods

Both groups were given routine treatment to improve circulation and nutritional nerves, and patients were instructed to use hypoglycemic drugs or control their blood sugar levels through insulin injection, to maintain fasting blood glucose of 4.0–7.6 mmol/L and blood glucose of 7.0–10.0 mmol/L at 2 h after meals.

### 2.4 CG

Conventional lipoic acid treatment was performed on the basis of conventional treatment. It selected 600 mg lipoic acid (manufacturer: Yantai Zhuchu Pharmaceutical Co., Ltd; national drug approval number H20080523; specification: 300 mg/piece), once a day, for a sequence treatment of 10 days, with a total of three courses of treatment [11].

### 2.5 EG

TCMP and red light therapy were implemented on the basis of the CG. TCMP was selected with the main ingredients (Safflower, *Angelica dahurica*, Qianghuo, Teasel root, Tanggu, *Angelica sinensis*, Sichuan pepper, Vinegar myrrh, Tougucao, Vinegar frankincense), and with 15 g of each drug dosage. After mixing the above drugs and developing them into powder, they were put into the TCMP. The patient was placed in a flat lying position and the TCMP was spread out in a flat manner on both lower limbs. Then, it was bound around the limbs in a circular shape for external application, with each treatment lasting for 30 min and treated twice a day. Red light therapy was implemented after applying TCMP externally. A red light therapy instrument was used (manufacturer: Beijing Aoer Huatai Technology Co., Ltd; model: HW-1000). First, it evaluated the specific range of the patient’s neuropathy, determined the specific number of electrode plates to be used, and selected four for most treatments. The determined electrode plates were comprehensively wrapped with disposable plastic. It was important to identify the acupoints such as the foot sanyang meridian and foot sanyin meridian in the lower limbs. The electrode plates were fixed on the acupoints. After turning on the instrument and adjusting the temperature, the patient was asked if their skin feels warm. The adjustment was done slightly to avoid burns. Treatment was conducted once a day for 30 min each time, with a duration of 10 days as a course of treatment for a total of three courses.

### 2.6 Observation indicators and evaluation

#### 2.6.1 General information

Based on the content of this study, the investigator formed a self-made general information questionnaire and evaluated it through medical record data review. The main content of the survey included patient gender, age, duration of DM, duration of DPN, HbAlc, FPG, TC, TG, HDL, and LDL. Blood glucose and lipid indicators were evaluated by taking fasting blood from patients and using laboratory biochemical instruments [12].

#### 2.6.2 Serum oxidation index level

The attending physician assisted the investigators to evaluate the serum malondialdehyde (MDA), erythrocyte superoxide dismutase (SOD), and total antioxidant capacity (TAOC) through the laboratory on the first day of admission and the day before discharge. The patients were advised to take 8 mL of venous blood on an empty stomach. The blood was placed in an anticoagulant tube, and centrifuged for 10 min at a speed of 3,000 r/min in a centrifuge, and then it was detected with enzyme linked immunosorbent assay and corresponding kits [13].

#### 2.6.3 Neuromotor and sensory conduction velocity

The attending physician assisted the investigator to use the electrophysiological parameter monitoring instrument (manufacturer: Shanghai Nuocheng Electric Co., Ltd; approval number: HSYJX (Z) Zi 2012 No. 2210115) on the first day of admission and the day before discharge to implement evaluation. The evaluation items included median nerve (MN), common peroneal nerve (CPN), and motor nerve conduction velocity (MNCV) [12].

#### 2.6.4 Neurological symptoms

The investigator was assisted by a primary nurse to conduct evaluations using the Toronto Clinical Scoring System (TSS) on the first day of admission and the day before discharge. The content assessed by the scale included the sensory abnormalities, pain, burning sensation, and numbness degree. Asymptomatic score was 0; occasional score was 0–3; frequent occurrence scores were 0, 1.33, 2.33, 3.33; and persistent presence scores were 0, 1.66, 2.66, 3.66. The total score range was 0–14.64, with higher scores indicating more severe symptoms of the disease. The coefficient value of scale Cronbach’s *α* was 0.867, and the internal consistency reliability was good.

#### 2.6.5 Statistical methods

The data were statistically processed using SPSS25.0, a dual entry software. The counting data indicators were described using case numbers (*n*) and rates (%), while the measurement data were expressed using mean ± standard deviation (*x* ± *s*). The chi square *x* ^2^ test and independent sample *t*-test were performed between groups. *P* < 0.05 denotes a statistically significant difference in data.

## 3 Results

### 3.1 Comparison of two sets of general information

It has no statistical significance difference in gender, age, duration of DM, duration of DPN, HbAlc, FPG, TC, TG, HDL, and LDL between the two groups (*P* > 0.05), as shown in Table 1.

### 3.2 Comparison of MDA, SOD, and TAOC levels between the two groups before treatment and after treatment

Before treatment, it has no statistical significance difference in the oxidation indicators between the two groups (*P* > 0.05). After treatment, the MDA in the two groups decreased, and the EG was lower than the CG, with a statistical significance difference (*P* < 0.05). The SOD and TAOC in both groups increased, and the EG was higher than the CG, with a statistically significant difference (*P* < 0.05), as shown in Table 2.

**Table 2:** Comparison of MDA, SOD, and TAOC levels between the two groups before treatment and after treatment (*x* ± *s*, points)

Group	Number of cases	MDA (mmol/L)		SOD (U/L)		TAOC (U/mL)	
		Before treatment	After treatment	Before treatment	After treatment	Before treatment	After treatment
CG	54	4.78 ± 1.34	3.64 ± 1.01*	46.34 ± 4.19	57.37 ± 5.09*	5.49 ± 0.75	8.34 ± 1.36*
EG	54	4.82 ± 1.26	3.21 ± 0.46*	46.67 ± 4.07	60.07 ± 5.47*	5.63 ± 0.81	9.75 ± 1.92*
*t*		0.160	2.847	0.415	2.655	0.932	2.530
*P*		0.873	0.005	0.679	0.009	0.354	0.013

Note: *Compared with before treatment in this group, *P* < 0.05; MDA: malondialdehyde; SOD: superoxide dismutase; TAOC: total antioxidant capacity.

### 3.3 Comparison of MNCV and SNCV index levels between the two groups before treatment and after treatment

Before treatment and after treatment, the MNCV and sensory nerve conduction velocity (SNCV) of the MN, CPN, and tibial nerve (TN) all increased, and the CG was lower than the EG, with a statistically significant difference (*P* < 0.05) and no comparison difference (*P* > 0.05). The details are denoted in Table 3.

**Table 3:** Comparison of MNCV and SNCV index between the two groups before treatment and after treatment (*x* ± *s*)

Group	Number of cases	MNCV (m/s)					
		MN		CPN		TN	
		Before treatment	After treatment	Before treatment	After treatment	Before treatment	After treatment
CG	54	42.35 ± 3.62	49.92 ± 3.94*	37.84 ± 2.63	42.17 ± 3.27*	36.45 ± 2.48	41.16 ± 2.97*
EG	54	42.70 ± 3.29	52.11 ± 4.30*	37.59 ± 2.28	44.20 ± 3.99*	36.72 ± 2.19	42.39 ± 3.15*
*t*		0.526	2.759	0.528	2.892	0.600	2.088
*P*		0.600	0.007	0.599	0.005	0.550	0.039

Group	Number of cases	SNCV (m/s)					
		MN		CPN		TN	
		Before treatment	After treatment	Before treatment	After treatment	Before treatment	After treatment
CG	54	41.55 ± 3.27	48.67 ± 4.12*	36.85 ± 2.06	43.15 ± 3.18*	40.11 ± 3.02	45.37 ± 3.64*
EG	54	41.73 ± 2.14	51.49 ± 4.36*	36.92 ± 2.17	45.27 ± 3.26*	40.25 ± 2.98	47.10 ± 3.93*
*t*		0.338	3.455	0.172	2.453	0.242	2.373
*P*		0.736	0.001	0.864	0.06	0.809	0.019

Note: *Compared with before treatment in this group, *P* < 0.05; MNCV: motor nerve conduction velocity; SNCV: sensory nerve conduction velocity.

### 3.4 TSS scores before treatment and after treatment in both groups

Before treatment, it has no statistical significance difference in TSS score between the two groups (*P* > 0.05). After treatment, the TSS score of both groups decreased, and the EG was lower than the CG, with a statistical significance difference (*P* < 0.05), as shown in Table 4.

**Table 4:** TSS score before treatment and after treatment in both groups (*x* ± *s*, points)

Group	Number of cases	TSS scores		*t*	*P*
		Before treatment	After treatment		
CG	54	11.34 ± 3.06	6.27 ± 2.14	9.978	0.000
EG	54	11.52 ± 3.18	4.34 ± 1.08	10.200	0.000
*t*		0.300	3.078		
*P*		0.765	0.003		

Note: TSS: Toronto Clinical Scoring System.

## 4 Discussion

The pathogenesis of DPN is closely related to the patient’s own age, duration of disease, high blood sugar, heredity, obesity, etc. Due to its complexity, clinical studies have not yet clarified the pathogenesis. It is analyzed that the disease may be caused by the combined effect of circulatory and metabolic disorders [14]. On the one hand, long-term high blood glucose and dyslipidemia are likely to lead to an activated state of multiple pathways in the cell, leading to inflammatory reaction of histiocyte and oxidative stress, thus damaging cell function and resulting in apoptosis [15]. On the other hand, endothelial cells and cells under the influence of blood sugar and blood lipids exhibit similar tissue pathological damage, leading to circulatory disorders, hypoxia, and ischemia of tissues, and causing serious damage to the structure and function of nerve cells. The above analysis is an important reference for clinical treatment of diseases. As an antioxidant, lipoic acid will not only reduce the lipid oxidation of nerve tissue, inhibit protein glycosylation, but also inhibit the activity of aldose reductase and the conversion of galactose or glucose, and it has a certain role in controlling blood sugar levels and improving symptoms [16]. The use of synthetic derivatives to treat peripheral neuropathy as a complication can have a significant impact on the quality of life of patients. Gholami et al. [17] analyzed the effect of zinc on chemotherapy-induced peripheral neuropathy (CIPN) using a double-blind control. The results showed that lipoic acid capsules could effectively reduce abnormal deep tendon reflex in patients, and there was a significant difference in activity limitation and pain severity compared to the CG (*P* < 0.001). Zinc supplementation therapy could reduce the frequency and intensity of CIPN chemotherapy. This result was correlated with the research finding that combination therapy can significantly reduce the TSS score of patients. The research results showed that after treatment, the MDA levels in the two groups decreased, and the EG was lower than the CG, with a statistically significant difference (*P* < 0.05). The levels of SOD and TAOC in both groups increased, and the EG was higher than the CG, with a statistically significant difference (*P* < 0.05). This result is consistent with the findings of Peng et al. Peng et al. [18] observed the effect of probucol combined with mecobalamin tablets on oxidative stress in patients with DPN. It was found that the TSS of patients after combined treatment decreased. SOD and CAT in the combined group were significantly higher than those in the CG (*P* < 0.05), and MDA was significantly lower than those in the CG (*P* < 0.05). Lipoic acid has the effect of improving insulin resistance, increasing blood flow in blood vessels, and effectively improving microcirculation and dilating blood vessels. Joint intervention methods can have a synergistic effect and improve treatment effectiveness. As a methylation active agent, methylcobalamin is more likely to enter nerve cells and can participate in the synthesis of methyltransferase nucleic acids, proteins, and phospholipids in the body through methylation function, thereby improving neurological function. Some studies have shown that mecobalamin combined with lipoic acid has a good application effect in the treatment of DPN, and the adjuvant therapy can effectively alleviate the clinical symptoms of patients [19].

Traditional Chinese medicine theory analysis shows that the main cause of the pathogenesis of DPN is prolonged thirst. Dryness and heat can lead to Yin damage and Qi depletion, ultimately resulting in a depletion of the body’s Qi, blood, and body fluids. Qi is the foundation of blood circulation, while Qi deficiency can lead to weakness in blood circulation, blood stasis in the limbs’ tendons and veins, and lack of nourishment in the tendons and veins. It will result in obstructed veins and inability to receive Qi and blood supply to the limbs, leading to symptoms and signs of neuropathy [20]. Therefore, it is necessary to supplement TCMP with western medicine in the DPN to improve the effectiveness of disease treatment. The effect of TCMP plus red light treatment combined with lipoic acid on the oxidative index in patients with DPN. MDA can mediate the oxidative damage of protein and lipid in the body, produce oxygen free radicals, and aggravate the oxidative stress damage of the body. It can lead to the destruction of the structure and function of neural microtubules, and can induce the mutation of neuraxis, and accelerate the apoptosis of nerve cells. SOD and TAOC can effectively eliminate oxygen free radicals and maintain the balance of body oxidation [21]. The drug effect of TCMP can protect the function of vascular endothelium, reduce the damage of vascular endothelium, expand blood vessels, and improve circulation. At the same time, it can make the peripheral nerve lesions to get sufficient blood oxygen supply, promote the metabolism of histiocyte, and improve the oxidation state of body tissues. Red light, as a photochemical reaction, can solidify proteins in diseased tissues in the shortest possible time. It can further accelerate blood circulation in local tissues, enhance immune function, improve tissue oxidation status, and optimize oxidation related indicators [22]. Sun et al. [23] and other studies show that red radiation can improve oxidative stress and inflammation and relieve pain by activating SPHK1/NF-κB in human keratinocytes.

Effects of TCMP plus red light therapy combined with lipoic acid on limb movement, sensory conduction function, and clinical symptoms of patients with DPN were analyzed. The research showed that, compared with lipoic acid alone, the combination of TCMP and red light irradiation-assisted lipoic acid treatment can significantly improve DPN’s symptoms of abnormal sensation, pain, burning, and numbness and the MNCV and SNCV of the MN, CPN, and TN, which is similar to Salvio AG’s research results [24]. The possible main reason is that TCMP treatment was implemented under the dialectical state of the overall body. By applying drugs to the lesion location of the lower limbs, the drugs could directly reach the lesion. In TCMP, the effects of Safflower and *Angelica sinensis* were to promote blood circulation, dissipate blood stasis, and reduce pain through meridians. The efficacy of *Angelica dahurica* was to dispel wind, remove dampness, reduce swelling, and relieve pain. The effects of Vinegar myrrh and Vinegar frankincense were to dispel wind, promote blood circulation, and relieve tendons and pain. The functions of Tougucao included promoting blood circulation, resolving blood stasis, and unblocking meridians and bones, while Qianghuo, Teasel root, and Tanggu had significant analgesic effects. The above important combination of external application had significant effects on promoting blood circulation and resolving blood stasis, warming meridians and alleviating pain, and promoting the recovery of clinical symptoms and signs in the limbs [25]. This result is similar to the research content of Ping. Among them, Jing et al. [26] evaluated the long-term efficacy of Astragalus-based traditional Chinese medicine in the treatment of DPN with the help of a randomized controlled trial. The results showed that the Astragalus-based traditional Chinese medicine group reduced the score of the TSS and serum interleukin-6, and the combination of traditional Chinese medicine and western medicine could increase the nerve conduction velocity, with good safety and efficacy.

In addition, TCMP promotes the absorption of drugs from the skin into the body through external application, which can promote the absorption of drugs in the lesion and the circulation of the tissue, alleviate the inflammatory response of the lesion, improve the symptoms of nerve hypoxia and ischemia and the state of cell damage, and promote the recovery of motor and sensory nerve conduction in the affected limbs [4]. Qiao et al. [27] also found that the external application of traditional Chinese medicine can effectively improve the treatment effect of patients with DPN, with an effective rate of more than 90%. Red light therapy, as a way of physical therapy of TCMP, can promote the release of nitric oxide from the hemoglobin of cells. After the body cells absorb nitric oxide, the capillaries of the diseased tissue will be expanded, further improving the local blood circulation of the tissue, and play the role of anti-inflammatory, analgesic, improving muscle spasm, and promoting tissue regeneration. Compared with lipoic acid alone therapy, TCMP combined with adjuvant therapy maximizes the effectiveness of drug therapy [28,29]. This result is highly consistent with the research approach of Lu et al. Chunjian et al. [30] used Bayesian network meta-analysis to evaluate the efficacy and safety of five commonly used combined traditional Chinese and western medicine external treatment methods for DPN. They found that foot bath, acupoint massage, acupoint injection, and moxibustion are superior to traditional western medicine alone in improving the motor conduction velocity of the MN, and their improvement effect on the SNCV of the CPN is better.

To sum up, in the treatment of patients with DPN with lipoic acid, the auxiliary TCMP plus red light treatment helps to inhibit oxidative stress reaction, promote cell metabolism and repair, improve limb movement and sensory function, and alleviate clinical symptoms and signs of disease.
